# Effects and Moderators of Triple P on the Social, Emotional, and Behavioral Problems of Children: Systematic Review and Meta-Analysis

**DOI:** 10.3389/fpsyg.2021.709851

**Published:** 2021-08-26

**Authors:** Na Li, Jin Peng, Yi Li

**Affiliations:** Department of Sociology, School of Humanities and Social Sciences, Xi'an Jiaotong University, Xi'an, China

**Keywords:** triple-p positive parenting program, social, emotional, behavioral problems, parenting, systematic review, meta-analysis

## Abstract

**Background:** Social, emotional, and behavioral problems in childhood are key predictors of persistent problem behaviors throughout the life courses of individuals. Early parental intervention training, as an important preventive measure, plays a critical role in improving the social, emotional, and behavioral (SEB) development of children.

**Method:** We conducted a systematic review and meta-analysis to analyze the intervention effects of the latest literature on Triple P (Positive Parenting Program), which is a multilevel system that provides treatment and prevention for children at risk of social, emotional, and behavioral problems *via* parenting approaches to enhance the parenting knowledge, skills, and confidence of parents. Since the literature on Triple P from 1970 to 2012 has already been systematically reviewed, this study searched the literature from 2013 to 2020 from the Web of Science, EBSCO, ERIC, MEDLINE, CNKI, and Triple P Evidence-Base website using multiple search strategies. This study differs from the existing research by its inclusion criteria of studies that use experimental designs or quasi-experimental designs. A total of 37 studies were included in the final analysis, and STATA 16.0 was used for evaluation while RevMan 5.3 for risk of bias assessment.

**Results:** The results show that Triple P can promote the social competence of children (*SMD* = 0.274) and prevent their emotional (*SMD* = −0.254) and behavioral problems (*SMD* = −1.38) to a certain extent. Simultaneously, the proximal effects on parents mainly included changing negative parenting styles (*SMD* = −0.46), reducing parenting conflicts (*SMD* = −0.311), and improving parenting efficacy and self-confidence (*SMD* = 0.419). The distal effects on parents included reducing the psychological adjustment problems of parents (*SMD* = −0.265), improving parent–child relationships, and reducing parent–child conflict (*SMD* = −0.714). However, the meta-analysis results did not show a significant effect of Triple P on improving the marital relationship quality and satisfaction of parents (*SMD* = 0.063). Components of the program intervention, including intervention level, service delivery format, service method, and program implementation setting, and the age of the children were crucial moderating factors on the outcomes of Triple P.

**Conclusion:** This study systematically reviewed the latest Triple P intervention literature and found the significant effectiveness of Triple P on the SEB problems of children and parenting outcomes and the moderators of the effect size.

## Introduction

The social, emotional, and behavioral (SEB) development of children is an essential topic to promote individual growth, family wellbeing, and social development (Sanders, 2012). This term refers to the social competence, emotional problems, and behavioral problems of children, and the definition of each part is: *social competence* is the ability of the children to use appropriate social skills in interactions with others and show pro-social and adaptive behaviors (Rantanen et al., 2012); *emotional problems* mean the negative emotions hidden inside that children fail to express or manage; *behavior problems* refer to the internalizing and externalizing behavioral issues of children, including withdrawal, refusal, and aggressive behaviors (Sanders et al., 2014). A latent transition analysis (LTA) by Basten et al. (2016) examined the stability of the behavioral problems of preschoolers and found that it had certain stability over time when children had co-occurring internalizing and externalizing problems. Thus, it would increase the risk of problem behaviors of the lives these children will have in the future. Evidence-based treatment was needed to prevent the adverse effects of the behavioral problems on the life courses of the individuals (Basten et al., 2016).

Parenting is a critical factor that affects the SEB development of children (Blader, 2006). Odgers et al. (2012) discovered that supportive parenting, especially maternal warmth, had a positively structural relationship with the social competence of children between 5 and 12 years, even under the circumstance of poverty in the United Kingdom. Maternal parenting stress would indirectly impact the social competence of children through parenting efficacy and behaviors, while positive maternal parenting behavior and a higher sense of efficacy could improve this social competence (Choi and Won, 2013). Simultaneously, negative parenting styles are risk factors that cause emotional and behavioral problems in children. Distant and excessively close parenting styles were both predictors of anxiety and depression in children aged 7–8 years old (Lindblom et al., 2017). A meta-analysis integrating 1,435 studies by Pinquart (2017) found that children showed higher externalizing problems when parents adopted negative parenting styles, such as harsh control or authoritarian, permissive, and neglectful parenting. In contrast, positive parenting styles, such as parental warmth, were protective factors that could reduce the behavioral problems of children (Hu and Jing, 2011; Li et al., 2016). The research about 10- to 13-year-old Palestinian children and their parents by Punamaki et al. (2017) proved that children who grew up in attached and warm family environments showed less internalizing, externalizing, and depressive problems.

Parenting interventions are essential for treating and preventing the problems of children and establishing a foundation to improve the wellbeing of children and families (Farrington and Welsh, 2003; McCart et al., 2006). In several countries, parenting interventions have both a rich history and an extensive evidence base. Barlow and Coren (2018) summarized six systematic reviews of parenting interventions published in the Campbell Library. These studies analyzed the effectiveness of different parenting styles in the primary and secondary prevention of the behavioral problems of children, treatment of conduct disorders in early childhood, and treatment of attention-deficit/hyperactivity disorder in children. The results showed that parenting intervention programs could not only enhance the mental health of parents but also effectively improve the emotional and behavioral adaptation of children.

Several parenting intervention programs are relatively mature, such as the meta-analysis results of two interventions, namely, the Parent–Child Interaction Therapy (PCIT) developed in the USA and the Positive Parenting Program (Triple P) developed in Australia, which have proven effective in reducing both the behavioral problems of 3- to 12-year-old children and the parenting problems of their caregivers (Thomas and Zimmer-Gembeck, 2007). Another systematic review of the Incredible Years (IY) Parenting Program has also been successful in improving disruptive behaviors and promoting the pro-social behaviors of children aged 3–9 in different families (Menting et al., 2013). These parenting intervention programs have an international influence and have the following common characteristics with other parent-focused intervention programs (Prinz, 2016): (1) they are founded in social learning theory and social interactional theory that both believe that interactions with family members are crucial factors in the development of behaviors in children, especially in preventing their misbehaviors; (2) they are action-centered behavioral parent training (BPT) interventions that focus on the specific parenting skills and strategies of parents rather than intervening directly with children; (3) they are problem-solving oriented, delivered in various content, formats, and modes, and widely spread. These interventions aim to prevent the problem behaviors of children, increase the parenting satisfaction and ability of parents, and strengthen the bond between children and parents. The vital role of parenting intervention programs in reducing and preventing the problems of children has been confirmed as they help parents reduce the internalization and externalization behaviors of children by improving parental cognition and emotional and behavioral regulation of parenting (Altafim et al., 2021).

Among these programs, Triple P has shown its distinguishing advantages because of its evidence-based features and popularity based on public health models (Liu and Guo, 2020). Triple P is a multilevel intervention strategy with various strengths, intensities, and scopes that focus on improving parenting. It provides treatment and prevention for children aged 0–16 years at risk of SEB problems by improving their knowledge, skills, and self-efficacy parents have in parenting and promoting safe and low-conflict environments for children. The key characteristics of Triple P are as follows:

(1) The target group is the parents (or caregivers with the role of a parent) of children aged 0–16 and includes children with varying degrees of behavioral and developmental problems, children at high risk of behavioral and developmental problems, and children in general.
(2) It contains five levels of interventions. The main difference lies in the severity of the “problem” of the target groups. The low-level interventions are universal and preventative for all groups or groups with low problem severity, while the high-level interventions are targeted at and therapeutic for more severely affected groups. Simultaneously, as the intervention level increases, the intensity of the intervention gradually increases. Level 1: Universal Triple P. It is a universal prevention intervention for all populations, and program coordinators use media and communication kits as the strategy to popularize acceptable parenting advice for parents and caregivers of children. It can also foster a supportive social environment for parents. Level 2: Selected Triple P. It is a brief parenting intervention that provides parenting information with a one- or two-session program to promote healthy development for specific subgroups that are more likely to face developmental problems. The tip sheets combined with video series are used to manage one or two discrete child behaviors. Level 3: Primary Care Triple P. It is a more intensive prevention strategy that targets parents who are concerned about the discrete problem behavior of their children and provides methods and skills to address specific issues with a four-session strategy. Level 4: Standard Triple P/Group Triple P/Self-Directed Triple P. It targets at-risk populations with problematic behaviors and children who meet diagnostic criteria to provide parents with early intervention and directed prevention to prevent the emergence of problematic behaviors and the development of severe impairments with an 8- to 10-session program. Meanwhile, the deficits in parenting skills of this kind of family are clear. Level 5: Enhanced Triple P. It targets families with additional risk factors that have not changed from the lower levels of intervention, in which children usually have severe behavioral problems and family dysfunction (Sanders and Prinz, 2005). This intervention in this level is highly targeted to the problem. The level of intervention is a crucial factor in moderating the effect of the intervention (Sanders et al., 2014).
(3) Unlike other intervention programs, Triple P is based on a public health model and is not limited to providing services only to vulnerable and high-risk families. It is also a preventive intervention measure that consists of a series of streamlined, low-cost, and different intervention levels, and has flexible delivery formats and service methods that can satisfy the fundamental needs of different parent groups across different childcare settings. The delivery formats include: face to face, online, face to face combined with telephone support, and online combined with telephone support. The service methods include: individual case, group, self-directed method, individual case combined with a group, and individual case combined with a self-directed method. They both vary according to the level of intervention correspondingly.
(4) The effectiveness of Triple P has been demonstrated by many evidence-based studies. Nowak and Heinrichs (2008) and Sanders et al. (2014) evaluated the effectiveness of Triple P in improving the parenting skills of parents and preventing SEB problems in children through systematic reviews. By comparing the information of the two studies in terms of the eligibility criteria, overall effect, and moderator effect, and by summarizing the findings, we can reveal that Triple P is a highly evidence-based parenting intervention program that can not only significantly prevent both SEB problems in children and negative parenting styles, but also effectively reduce parenting stress, enhance parenting efficacy, and improve the quality of parental relationships. At the same time, compared to other parenting intervention programs, Triple P has been widely replicated in at least 16 different countries (e.g., Australia, China, New Zealand, Turkey, Iran, and Indonesia) and diverse cultures, and has thus shown sound intervention effects and cultural adaptability (Heinrichs and Jensen-Doss, 2010; Hartung and Hahlweg, 2011). The country that implements the program does not influence the effect of the intervention, which means that the effectiveness of the intervention does not vary across countries and cultures, proving that Triple P has good cross-cultural adaptability.

In summary, many studies have shown that parenting skills are correlated with SEB problems in children, which are one of the main influencing factors and indicate that parenting intervention is a potential strategy for preventing and treating the problems of children. In recent years, a batch of increasingly mature parenting intervention programs has emerged, with such empirical studies supporting the critical role of these programs in promoting the wellbeing of parents and children. Among the many parenting intervention programs, Triple P has attracted attention owing to its good evidence-based cultural adaptability and confluence of prevention and treatment functions. Nowak and Heinrichs (2008) and Sanders et al. (2014) conducted comprehensive analyses of the literature on the effects of Triple P interventions published from 1970 to 2007 and 1980 to 2013, respectively, to demonstrate its effectiveness. In recent years, Triple P has been promoted and tested in a broader range of countries and settings, and new research results have been produced for different intervention levels and groups; thus, their effectiveness and related moderator effects need to be further evaluated and updated. Therefore, this study aims to systematically review the effectiveness of the Triple P intervention literature published from 2013 to 2020 in terms of the SEB problems of children and parenting outcomes.

## Method

Our study focused on the latest intervention effects of the Triple P intervention program on children and parents using a quantitative systematic review and a meta-analysis method. We collected Triple P intervention studies systematically according to specific inclusion/exclusion criteria, assessed the quality of evidence, designed information extraction forms, conducted data collection, and integrated data for analysis to summarize the effectiveness of Triple P in improving the SEB problems of children, parenting styles, parental adjustments, parental relationship satisfaction, and other aspects.

### Search Strategy

We searched for intervention research articles based on Triple P from different sources: the comprehensive databases including the Web of Science, EBSCO, MEDLINE, and CNKI, and thematic databases including ERIC and the Triple P Evidence-Base website of The University of Queensland (https://pfsc.psychology.uq.edu.au/research/triple-p-evidence-base) using “Triple P” and “intervention” as the search terms. The publication period was limited between January 2013 to December 2020 and the language was limited to Chinese or English. The Endnote 20.0 software was used to manage the articles. Three researchers were responsible for the screening and two more screened the literature independently according to the inclusion and exclusion criteria. If there were any differences of opinion, the literature was resolved through discussion or a consensus was reached through discussion with a third researcher.

### Inclusion/Exclusion Criteria

The inclusion and exclusion criteria in this study were formulated according to the PICOS approach; that is, (P) research population, (I) intervention measures, (C) comparison measures, (O) outcome indicators, and (S) study design. The specific standards are as follows:

(1) Population: The included studies should target the parents of the children. Regarding the definition of children, our study took the age range (0–16) of Triple P children as the same standard and included children with varying degrees of behavioral and developmental problems and children in general. Simultaneously, the included population must have parents as the primary intervention objects. Studies with “implementers” and “grandparents” as the intervention population should be excluded.
(2) Intervention: To ensure the independent effect of Triple P and eliminate interference from other interventions in the results, the included studies should include Triple P as the only intervention. Studies that combine other interventions should be excluded.
(3) Comparison: The comparison group should be a blank control group that does not receive interventions from Triple P or other parent training programs, which means that comparative studies on the effects of interventions between different forms of Triple P and comparative studies on the effects of interventions between Triple P and other interventions should be excluded.
(4) Outcomes: The included study should report at least one data point in the outcome category to be analyzed in the current study. The outcome category consists of the social competence, emotional problems, and behavioral problems of children, parenting styles, conflict over parenting, parenting confidence, parental adjustment, marital relationship quality and satisfaction of parents, and the parent–child relationship. Meanwhile, the included study must have sufficient data, including the mean and standard deviation of each outcome of the pre- and post-tests. Studies that only report results related to parents but not children will be excluded. Studies in which the scales used to measure outcomes differ significantly from conventional ones will also be excluded.
(5) Study design: The study design should be a randomized controlled trial (RCT) design or a quasi-experimental design.

### Type of Outcome

The characteristics of the outcomes were defined as follows (see the Appendix for specific measurement tools) to determine whether the effect value in the original study could be included in the extraction table: (1) social competence in children, which refers to the competence of children to interact and socialize with others, which includes, but is not limited to, the ability to express opinions and needs, ask for help, cooperate with the requirements of adults, get along with others, understand the feelings of others, understand the relationships between people, and to show a pro-social nature in interactions with others; (2) emotional problems in children, which may include anxiety, fear, depression, somatic complaints, shyness, and social withdrawal; (3) behavioral problems in children, which, based on the studies on the definition of the behavioral problems of children, we believe could include hyperactivity, disobedience, violation of discipline, and aggressive behavior (including problem and intensity); (4) parenting style, which refers to coping strategies used by parents to discipline their children, with parenting, according to our review of the existing literature, referring to a collection of attitudes and behaviors that parents convey to their children, and can be divided into different dimensions (including laxness, over-reactivity, and verbosity) using a conventional measurement tool; (5) conflict over parenting, which refers to disagreements and conflicts between parents in parenting, and mainly includes the problem and extent of parenting conflict; (6) parenting confidence, which refers to the self-evaluation of parents about their parenting ability, and mainly includes the sense of efficacy parents have for the behavior of their children and parenting settings; (7) parental adjustment, which refers to the pressure and adjustment of parenting and mainly includes depression, anxiety, and stress; (8) relationship quality and satisfaction of parents, which refers to the valuation and satisfaction of marital relationships between couples; (9) parent–child relationship, which evaluates the relationship between parents and children and is mainly reflected by parent–child conflict.

### Data Abstraction and Coding

Microsoft Excel was used to design the data extraction table and extract and manage the data. To ensure the accuracy of the data, two researchers extracted the information independently and then combined the data after re-checking and a consensus. Two types of data files were generated in our study: description and effect size files. The description item file included the following information: First, the basic information included the research title, publication type, publication time, name of the researchers, and data extraction date. Second, the sample characteristics included the average age and age range of children, gender ratio of children, initial symptoms/additional risks of children, severity of the initial problems of children, average age of parents, gender ratio of parents, sample characteristics at baseline (e.g., sample size of the total/intervention group/comparison group), the number of people lost to follow-up/withdrawal from the intervention and comparison groups, and the country and intervention setting. Third, the study design included extracted information on the comparison and the measures of intervention groups, such as level, version, form of service delivery, implementation time, frequency, duration, training of the intervention providers, and assessment information on the risk of bias. The effect size file concerned the extraction and sum of the relevant data for the outcomes and included nine sub-tables of the outcomes. In each sub-table, the measurement tools, reports, and intervention/control group data were recorded at the baseline and post-test time on the outcomes of each study, such as the number of participants, mean, standard deviation, and other data.

### Risk of Bias Assessment

The risk of bias within studies was assessed by two researchers independently according to the Cochrane Risk of Bias assessment tool (Cochrane RoB), which mainly included the generation of random sequences, concealment of the allocation scheme, blinding of participants and intervention practitioners, blinding of the outcome evaluator, integrity of the outcome data, risk of selective reporting of study results, and other sources of bias (Higgins and Green, 2008). They were assessed as “low,” “high,” and “unclear” in each study. We combined the data after re-checking and used RevMan 5.3 to assess the bias within the studies through the proportion of “low risk,” “high risk,” and “unclear” in each kind of bias. The publication bias was assessed by Egger's test (Egger et al., 2003).

### Statistical Model

The effect size was derived from the statistical standardization of initial results from different studies. It represented the quantitative results from a series of studies in a standardized form and allowed for meaningful numerical comparison and analysis between studies. In our study, the standardized mean difference (*SMD*) and its 95% confidence interval were used as statistics for the effect analysis (Yang et al., 2018). The random-effects model would be used unless there was only one study having the data of the outcomes we cared about. In the circumstance of only one study, we would have chosen the fixed-effect model (Borenstein et al., 2010).

Heterogeneity tests are essential in systematic reviews of social science as the differences among studies may lead to different research results. It is believed that, if the heterogeneity between the original studies is low, then the pooled effect size has high credibility. In this study, heterogeneity was tested using the *Q* test, *I*^2^ statistic (the variation in SMD attributable to heterogeneity), and the *Tau*^2^ statistic (the estimation of the between-study variance). If the result of the *Q* test is not significant (*p* > 0.05), it indicates that the heterogeneity between studies is low, and vice versa. The larger the *I*^2^ statistic, the higher the heterogeneity between the original studies is. When *I*^2^ < 50%, it means that there is a low to moderate degree of heterogeneity between the original research results, and when *I*^2^ ≥ 50%, there is a high degree of heterogeneity between the original research results (Yang et al., 2018). The larger the *Tau*^2^ statistic, the higher the heterogeneity between the original studies is (Borenstein et al., 2017). The effects were calculated and statistically analyzed using STATA 16.0.

### Subgroup and Meta-Regression Analyses

We conducted subgroup and meta-regression analyses on the outcomes with high heterogeneity. The sources of heterogeneity were the characteristics of the child, which mainly referred to whether the child had a developmental problem or an initial behavioral problem, and program characteristics, which included the format of service delivery, service method, level of Triple P, and program implementation environment including country and setting. Then, we performed a meta-regression analysis with child age, boy ratio, and sample size. We need to emphasize that, first, based on the conclusions of Nowak and Heinrichs (2008) and Sanders et al. (2014), we examined whether there were differences between the effects of Triple P among countries and its cultural adaptability. Second, children with developmental problems or behavioral problems, such as intellectual disability, attention-deficit/hyperactivity disorder, anxiety disorder, autism spectrum disorder, and behavioral problems as determined by measurement tools are more likely to have SEB problems than normal children. Therefore, we investigated whether there were differences in the effectiveness of Triple P for different groups, and attempted to verify whether Triple P was more effective in interventions for children with developmental problems and initial behavioral problems.

## Results

### Study Selection

**Figure 1:**
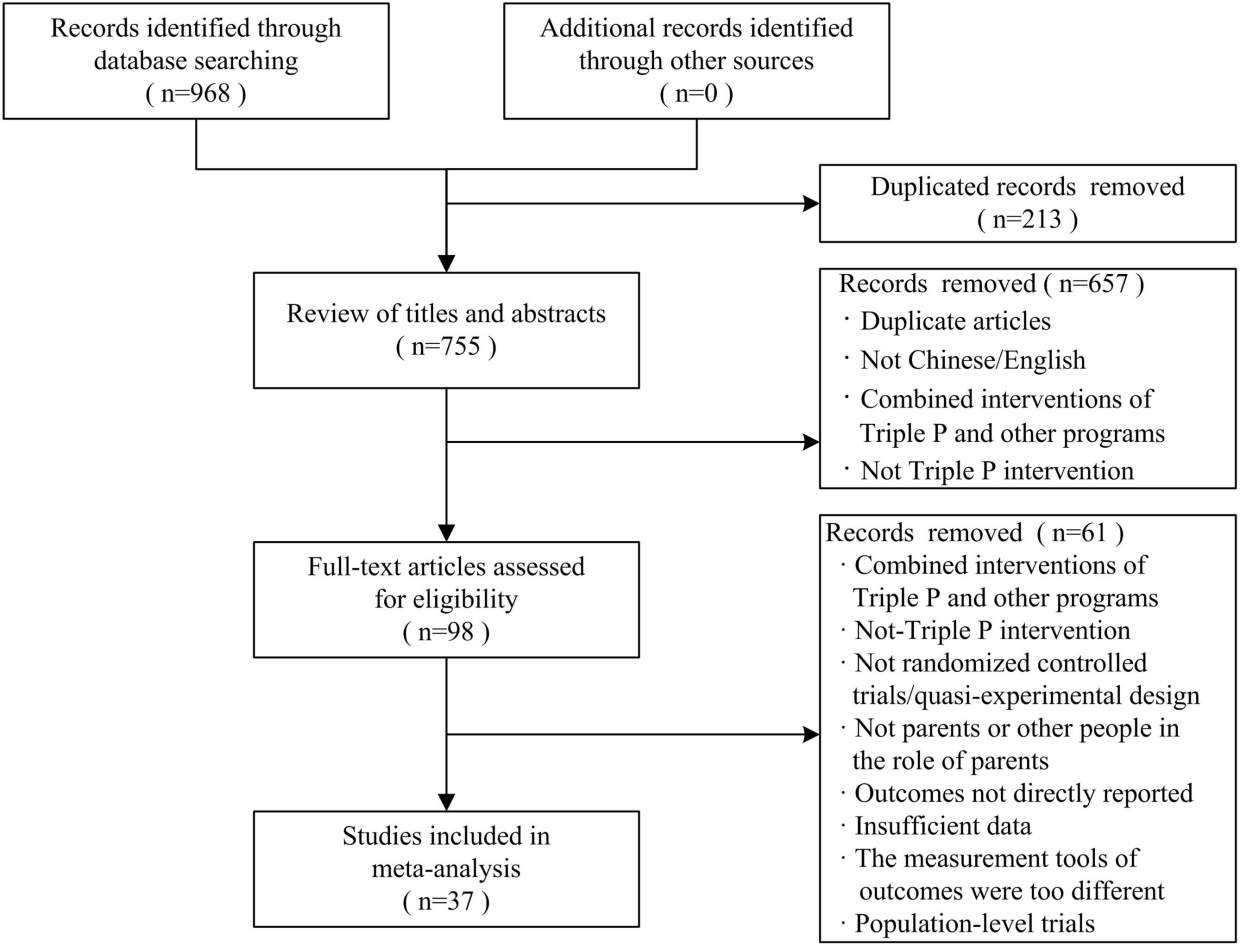
The process of the exclusion and the selection of studies in meta-analysis.

After the retrieval, 362 articles were from the Web of Science, 249 articles from EBSCO, 14 articles from MEDLINE and ERIC, and 343 articles from the Triple P Evidence-Base website. Finally, we obtained a total of 968 articles. Endnote 20.0 was used to remove duplicated articles, and we obtained a total of 755 articles. In the initial screening stage, two researchers (the first and third authors) excluded 657 unqualified articles according to their titles and abstracts based on the inclusion and exclusion criteria, which included duplicate articles, use of non-Chinese or English languages, combination of Triple P interventions with other programs, and non-Triple P intervention studies. Further, we re-screened the full text of 98 articles obtained after the initial screening for eligibility and excluded 61 articles. These articles included those that combined Triple P interventions with other programs, contained a non-randomized controlled experimental design or quasi-experimental design, had design and intervention objects that were not parents or caregivers with the role of a parent, had outcomes that were not directly reported, had insufficient data, had measurement tools of outcomes were too different, and had population-level changes. Finally, 37 articles were included in our meta-analysis. Figure 1 shows the search and screening processes used in our study.

### Study Characteristics

Table 1 provides basic information on the 37 studies. All the studies were conducted between 2013 and 2020, and 36 randomized controlled trials studies and one quasi-experimental study served 3,691 families (ranging from 17–355 families). The average age of children was 7.11 years (ranging from 2 to 17 years; SD = 4.62). The average proportion of boys was 61.9% (SD = 6.65). The children who received an intervention program in 28 studies had developmental or behavioral problems, including intellectual disability, attention-deficit/hyperactivity disorder, anxiety disorder, autism spectrum disorder, and behavioral problems as determined by measurement tools. In terms of the countries where programs were implemented, 16 were in Australia, 4 were in China, 3 were in New Zealand, 1 was in Australia and New Zealand simultaneously, and 13 were in other countries. Communities (n = 10) consisted of the main implementation setting. The other settings were healthcare centers, hospitals and clinics, schools, university health research centers, universities combined with communities, and online. In terms of the Triple P intervention levels, level 4 was the most common (*n* = 23), followed by levels 3, 5, and 2, in that order. There was no related research on the level 1 intervention in the included articles. The delivery formats of the interventions were “face-to-face,” “online,” “face-to-face combined with telephone support,” and “online combined with telephone support.” The service methods included the individual case, group, self-directed method, individual case combined with a group (*n* = 19), and individual case combined with a self-directed method. The average duration of the program intervention was 6.61 sessions.

**Table 1:** Study characteristics of the included studies.

**Sample characteristics**	**Sample size (N)**	**Sample characteristics**	**Sample size (N)**	**Other values**
**Country**		Face to face	14	
Australia	16	Online + telephone	3	
China	4	Online	3	
New Zealand	3	**Developmental/**		
Turkey	3	**Behavioral problems**		
Netherlands	3	Yes	28	
Iran	2	No	9	
Australia and New Zealand	1	**Level of Triple P**		
Indonesia	1	First level	0	
Ireland	1	Second level	1	
Panama	1	Third level	11	
Sweden	1	Fourth level	23	
the UK	1	Fifth level	2	
**Setting**		**Service methods**		
Community	10	Case	4	
Hospital and clinic	6	Group	9	
School	5	Self-directed	3	
University	5	Case + group	19	
Online	5	Case + self-directed	2	
Health care center	4	**Child age**		7.11 (year)
University and community	2	**Average % of boy**		61.9 (%)
**Delivery format**		**Average session**		6.61 (session)
Face to face+telephone	17	**Total sample size**		3691 (family)

*Bold value indicate dimensions of study characteristics*.

### Overall Effect Size

Table 2 summarizes the effects of Triple P on the nine outcomes and other statistics, and includes the number of articles that reported the outcomes (*k*), pooled *SM*D, 95% confidence interval (*CI*), *I*^2^, *Tau*^2^, and *Tau*.

**Table 2:** The overall effect size of Triple P on the outcomes.

	***k***	***Pooled SMD***	***Lower 95% CI***	***Upper 95% CI***	***I^**2**^***	***Tau^**2**^***	***Tau***
1. Social competence in child	6	0.274	0.025	0.523	0.0%	0.000	0.000
2. Emotional problems in child	13	−0.254	−0.476	−0.031	65.0%	0.102	0.319
3. Behavioral problems in child-Total	17	−1.380	−2.161	−0.599	97.3%	2.508	1.584
3.1 Problem	15	−0.467	−0.631	−0.303	42.5%	0.042	0.205
3.2 Intensity	17	−0.378	−0.501	−0.255	15.6%	0.010	0.100
4. Parenting Style-Total	18	−0.460	−0.717	−0.203	83.3%	0.249	0.499
4.1 Laxness	15	−0.482	−0.653	−0.310	50.9%	0.056	0.237
4.2 Overreactivity	15	−0.480	−0.645	−0.315	47.3%	0.048	0.219
4.3 Verbosity	10	−0.639	−0.878	−0.399	59.6%	0.084	0.290
5. Conflict over Parenting-Total	6	−0.311	−0.515	−0.106	11.4%	0.008	0.089
5.1 Problem	8	−0.333	−0.638	−0.029	69.0%	0.127	0.356
5.2 Extent	9	−0.268	−0.511	−0.025	57.8%	0.076	0.276
6. Parenting Confidence-Total	28	0.419	0.309	0.530	32.1%	0.027	0.164
6.1 Behavior	14	0.491	0.339	0.643	34.3%	0.028	0.167
6.2 Setting	9	0.304	0.111	0.497	32.1%	0.028	0.167
7. Parental Adjustment-Total	15	−0.265	−0.402	−0.128	19.3%	0.014	0.118
7.1 Depression	15	−0.571	−1.003	−0.139	94.3%	0.660	0.812
7.2 Anxiety	14	−0.278	−0.427	−0.128	48.7%	0.034	0.184
7.3 Stress	14	−0.582	−1.055	−0.108	95.0%	0.748	0.865
8. Parent-Child relationships	5	−0.714	−1.292	−0.135	85.2%	0.368	0.607
9. Relationship Satisfaction of parents	9	0.063	−0.228	0.354	64.4%	0.126	0.355

*k, number of studies; SMD, standard mean difference; CI, confidence interval; I^2^, the variation in SMD attributable to heterogeneity; Tau^2^, the estimation of the between-study variance*.

#### SEB Problem Outcomes in Children

Six intervention studies reported results relating to the social competence of children and 13 reported results relating to the emotional problems of children. We found significant differences in the outcomes of the social competence [*SMD* = 0.274, *95% CI* (0.025, 0.523)] and emotional problems [*SMD* = −0.254, *95% CI* (−0.476, −0.031)] of children between the experimental and control groups, showing that Triple P can significantly improve the social competence of children while reducing their emotional problems. Seventeen intervention studies reported results relating to the behavioral problems of children. The pooled *SMD* of the behavioral problems of children was −1.38 and its 95% confidence interval ranged from −2.161 to −0.599. Therefore, we can conclude that the Triple P intervention can significantly reduce the behavioral problems of children.

Fifteen studies reported the effects of Triple P on the number of behavioral problems of children. The pooled *SMD* was −0.467 and its 95% confidence interval ranged from −0.631 to −0.303, showing that the experimental and control groups had significant differences in the number of the behavioral problems of children, which indicates that Triple P can significantly reduce this number. Seventeen studies reported the effect of Triple P on the intensity of behavioral problems. The pooled *SMD* was −0.378 with a 95% confidence interval that ranged −0.501 to −0.255, indicating that there was a significant difference between the experimental and control groups. Therefore, Triple P can significantly reduce the intensity of behavioral problems in children.

#### Parental Outcomes: Proximal Effects

The proximal effects mainly included *parenting style, conflict over parenting*, and *parenting confidence*. For parenting style, the outcomes reported in the original research consisted of four types: total score of *parenting styles* [*SMD* = −0.46, *95% CI* (−0.717, −0.203)] and the score of the sub-dimensions of *laxness* [*SMD* = −0.482, *95% CI* (−0.653, −0.31)], *over-reactivity* [*SMD* = −0.48, *95% CI* (−0.645, −0.315)], and *verbosity* [*SMD* = −0.639, *95% CI* (−0.878, −0.399)]. The results showed that the experimental and control groups had significant differences in these four dimensions, indicating that Triple P can significantly prevent negative parenting styles in parents. The analysis of *conflict over parenting* included three aspects: *total score* [*SMD* = −0.311, *95% CI* (−0.515, −0.106)], *problem* [*SMD* = −0.333, *95% CI* (−0.638, −0.029)], and *extent* [*SMD* = −0.268, *95% CI* (−0.511, −0.025)]. The meta-analysis results showed significant differences between the experimental and control groups in these dimensions, indicating that Triple P can significantly reduce conflict over parenting. The analysis of parenting efficacy also included three aspects: *total score* [*SMD* = 0.419, *95% CI* (0.309, 0.53)] and the sub-dimensions of *behavior self-efficacy* [*SMD* = 0.491, *95% CI* (0.339, 0.643)] and *setting self-efficacy* [*SMD* = 0.304, *95% CI* (0.111, 0.497)]. The meta-analysis results showed that there were significant differences between the experimental and control groups regarding the changes in the above data, showing that Triple P can significantly improve parenting efficacy.

#### Parental Outcomes: Distal Effects

The distal effects mainly included *parental adjustment, parent–child relationship*, and *marital relationship quality and satisfaction of parents*. For *parental adjustment*, in addition to the *parental adjustment* total score, the original studies also reported the scores of the three sub-dimensions of *depression, anxiety*, and *stress*. The results showed that the experimental and control groups had different total scores for *parental adjustment* [*SMD* = −0.265, *95% CI* (−0.402, −0.128)], *depression* [*SMD* = −0.571, *95% CI* (– 1.003, −0.139)], *anxiety* [*SMD* = −0.278, *95% CI* (−0.427, −0.128)], and *stress* [*SMD* = −0.582, *95% CI* (−1.055, −0.108)], indicating that Triple P can significantly improve the level of psychological adjustment of parents. In addition, the meta-analysis results showed that Triple P had a significant effect on reducing parent–child conflict [*SMD* = −0.714, *95% CI* (−1.292, −0.135)], but we did not find a significant effect of Triple P on the marital relationship quality and satisfaction of parents [*SMD* = 0.063, *95% CI* (−0.228, 0.354)].

### Heterogeneity Sources and Moderating Effects

Table 3 shows the results of subgroup analysis. Table 4 shows the results of meta-regression analysis.

**Table 3:** The results of the subgroup analysis.

**Outcomes**	**Sources of heterogeneity**	**Subgroup differences**	**Categories**	***k***	***pooled SMD***	***lower 95% CI***	***upper 95% CI***	***z***	***P***
Emotional problems in children	Developmental/ Behavioral problems	*P = 0.02*	Yes	9	−0.355	−0.733	0.023	1.84	0.066
			No	4	−0.314	−0.565	−0.062	2.45	0.014*
	Level of Triple P	*P < 0.001*	Third level	4	0.088	−0.129	0.304	0.79	0.427
			Fourth level	8	−0.612	−0.946	−0.278	3.59	0.000^***^
	Delivery format	*P < 0.001*	Face to face + telephone	4	−0.761	−1.040	−0.481	5.34	0.000^***^
			Face to face	6	−0.234	−0.654	0.186	1.09	0.274
			Online	2	−0.151	−0.714	0.411	0.53	0.598
	Service method	*P < 0.001*	Group	5	−0.230	−0.722	0.262	0.92	0.359
			Self-directed	2	−0.151	−0.714	0.411	0.53	0.598
			Group + case	4	−0.761	−1.040	−0.481	5.34	0.000^***^
	Country	*P < 0.001*	Australia	8	−0.221	−0.563	0.121	1.27	0.205
			Other countries	4	−0.529	−1.019	−0.040	2.12	0.034^*^
Behavioral Problems in Children	Level of Triple P	*P < 0.001*	Third level	3	0.143	−0.304	0.590	0.63	0.531
			Fourth level	12	−0.758	−1.184	−0.332	3.49	0.000^***^
	Delivery format	*P < 0.001*	Face to face + telephone	8	−3.004	−4.721	−1.288	3.43	0.001^***^
			Face to face	7	−0.408	−1.022	0.206	1.30	0.193
			Online + telephone	2	−0.015	−0.400	0.370	0.08	0.938
	Service method	*P < 0.001*	Case	3	−0.017	−0.603	0.569	0.06	0.956
			Group	4	−0.695	−1.618	0.228	1.48	0.14
			Group + case	9	−2.676	−4.267	−1.086	3.3	0.001^***^
	Setting	*P < 0.001*	Community	2	−0.377	−0.931	0.178	1.33	0.183
			Hospital and clinic	6	−3.928	−6.437	−1.419	3.07	0.002^**^
			University	4	−1.055	−1.751	−0.359	2.97	0.003^**^
			Online	2	−0.015	−0.400	0.370	0.08	0.938
			Health care center	2	0.203	−0.536	0.941	0.54	0.590
Parenting	Level of Triple P	*P < 0.001*	Third level	6	0.013	−0.381	0.407	0.06	0.949
Style			Fourth level	10	−0.615	−0.855	−0.376	5.04	0.000^***^
	Delivery format	*P < 0.001*	Face to face + telephone	7	−0.917	−1.208	−0.626	6.18	0.000^***^
			Face to face	8	−0.088	−0.404	0.227	0.55	0.584
			Online + telephone	2	−0.443	−0.737	−0.150	2.96	0.003^**^
	Service method	*P < 0.001*	Case	2	0.468	−0.611	1.547	0.85	0.395
			Group	6	−0.287	−0.503	−0.072	2.62	0.009^**^
			Group + case	7	−0.917	−1.208	−0.626	6.18	0.000^***^
			Self-directed + case	2	−0.443	−0.737	−0.150	2.96	0.003^**^
	Setting	*P < 0.001*	Community	4	−0.322	−0.554	−0.090	2.72	0.007^**^
			Hospital and clinic	4	−0.703	−1.344	−0.061	2.15	0.032^*^
			University	2	−0.609	−0.907	−0.312	4.01	0.000^***^
			Online	3	−0.528	−0.775	−0.281	4.19	0.000^***^
			Health care center	2	0.468	−0.611	1.547	0.85	0.395
			University and community	2	−0.515	−1.419	0.388	1.12	0.264
Conflict over	Level of Triple P	*p = 0.13*	Third level	3	−0.363	−1.085	0.358	0.99	0.324
Parenting			Fourth level	5	−0.349	−0.645	−0.052	2.31	0.021^*^
	Setting	*P = 0.08*	Community	4	−0.478	−0.947	−0.009	2.00	0.046^*^
Parent-Child			Online	2	−0.205	−0.854	0.444	0.62	0.535
Relationship	Level	–	Fourth level	4	−0.904	−1.528	−0.280	2.84	0.005^**^

*We conducted a subgroup analysis of the outcomes with high heterogeneity (Q test is significant, p < 0.05 and I^2^ ≥ 50%). The results of subgroup analysis of only one study were not presented in this Table. k, number of studies; SMD, standard mean difference; CI, confidence interval*;

* *p < 0.05*

** *p < 0.01*.

*** *p < 0.001*.

**Table 4:** The results of the meta-regression analysis.

**Outcomes**	**Moderators**	**k**	**Coef**.	**Std. Err**.	**t**	**P>|t|**	**[95% Conf. Interval]**	
Emotional problems	Child age	13	−0.121	0.043	2.80	0.017^*^	−0.216	−0.026
in children	Boy ratio	13	−0.774	1.688	−0.46	0.655	−4.488	2.940
	Sample size	13	0.006	0.003	2.08	0.062	−0.000	0.012
Behavioral problems in	Child age	17	0.009	0.322	0.03	0.978	−0.687	0.705
Children	Boy ratio	17	2.693	9.692	0.28	0.785	−18.245	23.631
	Sample size	17	−0.013	0.015	−0.88	0.396	−0.046	0.019
Parenting style	Child age	18	0.034	0.047	0.73	0.476	−0.066	0.135
	Boy ratio	18	−0.174	1.338	−0.13	0.898	−3.042	2.695
	Sample size	18	−0.002	0.002	−0.73	0.478	−0.006	0.003
Conflict over parenting	Child age	8	0.068	0.039	1.77	0.151	−0.039	0.176
	Boy ratio	8	2.510	0.963	2.60	0.060	−0.165	5.184
	Sample size	8	0.004	0.003	1.14	0.300	−0.004	0.012
Parent-child relationship	Child age	5	−0.138	0.314	−0.44	0.736	−4.123	3.847
	Boy ratio	5	−4.915	7.626	−0.64	0.636	−101.819	91.988
	Sample size	5	0.030	0.037	0.83	0.558	−0.423	0.482

*k, number of studies; Coef., coefficient; Std. Err., standard error; 95% Conf. Interval, 95% confidence interval*;

* *p < 0.05*.

#### Subgroup and Meta-Regression Analyses of Emotional Problems in Children

According to the subgroup analysis results of emotional problems in children, we could first conclude that the presence of a developmental/behavioral problem, the level of Triple P, service delivery format, service method, and country of the intervention implementation were all heterogeneity sources of the emotional problems of children. The differences of the pooled effect sizes between the subgroups were all statistically significant (most *p < 0.001*). First, in terms of the sample characteristics, the effect of Triple P on reducing the emotional problems in children without developmental or behavioral problems was significant [*SMD* = −0.314, *95% CI* (−0.565, −0.062)], which was contrary to our expectations. Second, interventions in other countries [*SMD* = −0.529, *95% CI* (−1.019, −0.04)] had a stronger effect on the outcomes of emotional problems in children than interventions in Australia [*SMD* = −0.221, *95% CI* (−0.563, 0.121)]. Third, regarding the components of the intervention, level 4 [*SMD* = −0.612, *95% CI* (−0.946, −0.278)] showed significant effects in reducing the emotional problems of children. Correspondingly, the “face-to-face combined with telephone support” format [*SMD* = −0.761, *95% CI* (−1.04, −0.481)] and the “group activities combined with individual case counseling” methods [*SMD* = −0.761, *95% CI* (−1.04, −0.481)] were both significant. We also conducted a meta-regression analysis with child age, boy ratio, and sample size as the moderating variables. The results showed that child age (β = −0.121, *p* < 0.05) was negatively correlated with emotional problems, while the effects of boy ratio (β = −0.774, *p* > 0.05) and sample size (β = 0.006, *p* > 0.05) on emotional outcomes were not significant. This indicates that, the older the child, the stronger the effect in the reduction of emotional problems in children. The boy ratio and the sample size of the intervention did not have significant effects on the emotional problems of the children.

#### Subgroup Analysis of Behavioral Problems in Children (Total Score)

We conducted a subgroup analysis of behavioral problems in children and found that the presence of a developmental/behavioral problem, country, age of the children, boy ratio, and sample size did not significantly moderate the outcomes and were not sources of heterogeneity. These contrast with the findings of Sanders et al. (2014), who found that country, presence of a developmental/behavioral problem, and child age were significant moderators of SEB in children. The heterogeneity of behavioral problems in children came from four sources, namely, Triple P level, service delivery format, service method, and intervention implementation setting, and the differences of the pooled effect sizes between the subgroups were all statistically significant (*p < 0.001*). First, in terms of the intervention levels of Triple P, level 4 [*SMD* = −0.758, *95% CI* (−1.184, −0.332)] could significantly reduce behavioral problems in children, while the effect of level 3 was not significant [*SMD* = 0.143, *95% CI* (−0.304, 0.59)]. Second, regarding the format of service delivery, “face-to-face activities combined with telephone support” [*SMD* = −3.004, *95% CI* (−4.721, −1.288)] showed a significant effect, while “face-to-face activities” [*SMD* = −0.408, *95% CI* (−1.022, 0.206)] and “online combined with telephone support” [*SMD* = −0.015, *95% CI* (−0.4, 0.37)] had no significant effects. In terms of the service methods, “group activities combined with case counseling” [*SMD* = −2.676, *95% CI* (−4.267, −1.086)] showed strong effect, while group [*SMD* = −0.695, *95% CI* (−1.618, 0.228)] and case counseling [*SMD* = −0.017, *95% CI* (−0.603, 0.569)] methods did not explain the heterogeneity between the effect sizes significantly. Interventions in hospitals and clinics [*SMD* = −3.928, *95% CI* (−6.437, −1.419)] and universities [*SMD* = −1.055, *95% CI* (−1.751, −0.359)] were significantly effective, in addition to when implemented in both universities and communities simultaneously. The effect of the intervention implemented in community [*SMD* = −0.377, *95% CI* (−0.931, 0.178)] alone was non-significant, while the effects of online [*SMD* = −0.015, *95% CI* (−0.4, 0.37)] and healthcare centers [*SMD* = 0.203, *95% CI* (−0.536, 0.941)] were not significant.

#### Subgroup Analysis of Parenting Styles

Consistent with the subgroup analysis results of problem behaviors in children, the subgroup analysis results of parenting styles showed that the presence of a development/behavioral problem, country, age of children, boy ratio, and sample size did not explain the heterogeneity between the effect sizes significantly. Heterogeneity came from four sources: Triple P level, service delivery format, service method, and implementation setting. The differences of the pooled effect sizes between the subgroups were all statistically significant (*p < 0.001*). In terms of the intervention levels of the Triple P, level 4 [*SMD* = −0.615, *95% CI* (−0.855, −0.376)] could significantly improve parenting style, while the effect of level 3 [*SMD* = 0.013, *95% CI* (−0.381, 0.407)] was not significant. In the service delivery format, only “face-to-face activities” [*SMD* = −0.088, *95% CI* (−0.404, 0.227)] was non-significant. “Face-to-face activities combined with telephone support” [*SMD* = −0.917, *95% CI* (−1.208, −0.626)] and “online combined telephone support” [*SMD* = −0.443, *95% CI* (−0.737, −0.15)] were both effective. “Face-to-face case counseling” [*SMD* = 0.468, *95% CI* (−0.611, 1.547)] was non-significant, but “face-to-face group activities” [*SMD* = −0.287, *95% CI* (−0.503, −0.072)], “group activities combined with case counseling” [*SMD* = −0.917, *95% CI* (−1.208, −0.626)], and “self-directed combined case counseling” [*SMD* = −0.443, *95% CI* (−0.737, −0.15)] were all effective. Interventions in hospitals [*SMD* = −0.703, *95% CI* (−1.344, −0.061)], communities [*SMD* = −0.322, *95% CI* (−0.554, −0.09)], universities [*SMD* = −0.609, *95% CI* (−0.907, −0.312)], and online [*SMD* = −0.528, *95% CI* (−0.775, −0.281)] were all effective. It did not show significant effects when interventions were implemented in healthcare centers [*SMD* = 0.468, *95% CI* (−0.611, 1.547)] and universities in combination with communities [*SMD* = −0.515, *95% CI* (−1.419, 0.388)].

#### Subgroup Analysis of Conflict Over Parenting

In the subgroup analysis of conflict over parenting, the level of Triple P and implementation setting were sources of heterogeneity. The subgroup analysis results showed that level 4 of the Triple P [*SMD* = −0.349, *95% CI* (−0.645, −0.052)] had a significant effect on reducing the conflict over parenting of parents. The community [*SMD* = −0.478, *95% CI* (−0.947, −0.009)] was the only setting showing a significant effect on reducing the conflict over parenting of parents. However, neither of the differences of the pooled effect sizes between the subgroups were significant (*p* = *0.13 and p* = *0.08*). Further research based on more studies is needed.

#### Subgroup Analysis of Parent–Child Relationship

The heterogeneity of the parent–child relationship came from the level of Triple P. The level 4 intervention [*SMD* = −0.904, *95% CI* (−1.528, −0.280)] had a significant effect in reducing the parent–child relationship. As the other subgroups included only one study, this conclusion was not accurate. We need more studies for further research.

### Risk of Bias

#### Risk of Bias Within Studies

**Figure 2:**
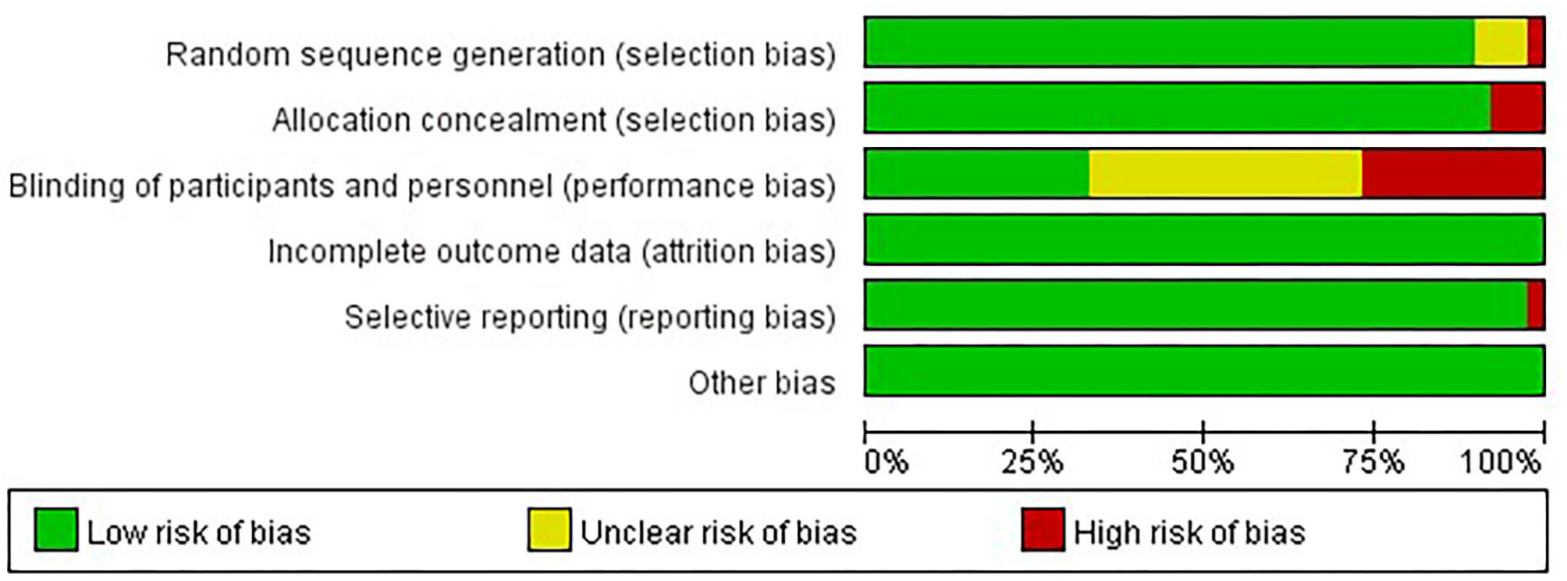
The results of the risk of bias within studies.

Figure 2 shows the results of the risk of bias within studies. In terms of the selection bias, the high-risk sequence generation accounted for a relatively small proportion. The generation of the random sequence had a low risk. None of the allocation concealments of the studies were unclear. There were a small number of studies that had not concealed its random allocation, with most of the studies carried out allocation concealment. The highest risk of bias was performance bias. Attrition bias in all studies was low risk, while the reporting bias was relatively low. Only one study reported incomplete results that differed from the data expected to be reported in the study design. To summarize, the risk bias within studies is low, except for the performance bias.

#### Publication Bias

We used Egger's test to assess the publication bias (see Table 5). The results showed that most of the outcomes of the children and parents were not significantly affected by the publication bias, except for the emotional problems of children and parental parenting confidence. Due to the limited number of included studies, the small sample size may lead to a certain degree of bias.

**Table 5:** Publication bias.

	**k**	**Coef**.	**Std. Err**.	**t**	**P>|t|**	**[95% Conf. Interval]**	
Publication bias of social competence	6	−1.402	1.698	−0.83	0.455	−6.116	3.311
Publication bias of emotional problems	13	−3.238	1.385	−2.34	0.039^*^	−6.288	−0.189
Publication bias of behavioral problems	17	−8.938	4.281	−2.09	0.054	−18.063	0.188
Publication bias of parenting styles	18	2.513	2.193	1.15	0.269	−2.135	7.161
Publication bias of conflict over parenting	6	−3.224	1.471	−2.19	0.094	−7.309	0.861
Publication bias of parenting confidence	14	3.158	1.169	2.70	0.019^*^	0.611	5.706
Publication bias of parental adjustment	15	−2.328	1.127	−2.07	0.059	−4.762	0.105
Publication bias of parent-child relationship	5	−12.162	5.540	−2.20	0.116	−29.793	5.469
Publication bias of relationship quality and satisfaction of parents	9	3.427	3.722	0.92	0.388	−5.373	12.228

*k, number of studies; Coef., coefficient; Std. Err., standard error; 95% Conf. Interval, 95% confidence interval*;

* *p < 0.05*.

## Discussion

This study searched and screened Triple P intervention studies published from 2013 to 2020 and synthesized the results using a meta-analysis and a systematic review. We searched a total of 968 studies and included 37 articles for meta-analysis. These studies all published high-quality and rigorously designed RCT studies. Thus, our research conclusions could rigorously evaluate the effectiveness of Triple P.

In terms of the overall effect of Triple P, the meta-analysis results showed that Triple P could provide a series of differences between children and parents, including the significant prevention of SEB problems in children. The proximal effects on parents mainly included changing negative parenting style, reducing parenting conflict, and improving parenting efficacy and self-confidence. The distal effects on parents included reducing psychological adjustment problems in parents, improving parent–child relationships, and reducing parent–child conflict. However, the meta-analysis results did not show the significant effects of Triple P on improving the marital relationship quality and satisfaction of parents.

Regarding the moderator variables, we conducted subgroup and meta-regression analyses of the effect sizes with high heterogeneity. The five groups of analysis results showed that components of the intervention, including intervention level, service delivery format, service method, and program implementation setting, explained the heterogeneity between the effect sizes of Triple P significantly, as follows: First, the sessions and intensities were different for the different Triple P levels, so their outcomes varied. Combined with previous research, we believe that the higher the intervention level, the better the effect. In our results, the level 3 intervention did not show significant effectiveness in the subgroup analysis, while the level 4 intervention had significant effectiveness in improving emotional problems in children, behavioral problems in children, parenting styles, parental conflict, and the parent–child conflict, which is consistent with the research conclusion of De Graaf et al. (2008). This finding contrasts with Sanders et al. (2014), who found that all Triple P levels had significant amounts of heterogeneity for all outcomes. In addition, since the Triple P levels generally corresponded to the program variants, the number of articles included in our study was limited as there were many variants of Triple P, and the specific analysis of the program variants might lead to deviations in the results. This differed from Sanders et al. (2014) in that we did not analyze the moderating effect of the program variants.

Second, regarding the service delivery formats and service methods, based on the five different delivery formats of Triple P classified by Sanders (2012), we distinguished the service delivery formats and service methods and conducted further in-depth analysis. Different service delivery formats and corresponding service methods led to different outcomes. Although our research did not discover consistent and effective service delivery formats and service methods for all outcomes, we can still conclude that we can achieve excellent parent-related outcomes through various formats and methods (especially in the improvement of parenting styles). However, to achieve good children-related outcomes, although the different intervention levels have a significant impact, we still need to adopt specific formats and methods, such as face-to-face group activities combined with telephone support for individual cases. This is because Triple P intervenes directly with parents. To further reduce SEB problems in children by improving parenting styles, more intensive service delivery formats and service methods are needed. Meanwhile, in contrast with Sanders et al. (2014), we found that the online format only showed significant effect on the improvement of parenting styles, but we did not verify the overall higher effect sizes for online Triple P as Sanders et al. (2014) did. We must be extra cautious when choosing an online method.

Third, regarding the countries where Triple P interventions were implemented, the results proved the significant effectiveness of Triple P in other countries in terms of the emotional problems in children. However, considering that there were only four interventions in other countries, this result needs to be further verified in future studies. More interventions and more evidence of Triple P in countries that have fewer studies available than Australia are needed. In addition, the meta-analysis results did not reveal country-to-country differences in the effects of Triple P on most outcomes of parents and children, which further proves the cultural adaptability of Triple P. In terms of the implementation settings of Triple P, the university was a vital setting which had a significant impact on the results in all subgroup analyses. In addition, interventions implemented in the community alone had no significant effect on the outcomes, but when implemented in both universities and communities simultaneously, the effectiveness of the outcomes became significant. This may be because the scientific research and professional specialties in universities can implement and standardize the program more rigorously, thereby improving the effectiveness of the interventions. In addition, hospitals, communities, and online were also settings with high significance, but the interventions implemented in healthcare centers had no significant effect. In terms of the other sample characteristics, we found the moderating effect of the age of children on emotional problems in children but did not discover the significant moderating role of the initial developmental/behavioral problems or boy ratio on the outcomes of children and parents, which differed from Sanders et al. (2014) who found higher effect sizes in children with initial developmental/behavioral problems.

Fourth, in terms of the risk of bias within studies, all RCT studies could not make participants blind to the intervention they received, which is consistent with other psychological intervention studies. Therefore, performance bias may have a certain impact on the results of data analysis.

## Conclusion

This study systematically reviewed the Triple P intervention literature published from 2013 to 2020 and found the significant effectiveness of Triple P in terms of preventing SEB problems in children and improving parenting outcomes, as well as the moderators of the effect size. We can conclude that: first, level 4 Triple P is widespread and the most effective on the SEB problems in children and parenting skills of parents; second, Triple P intervenes directly with parents, thus, to further prevent SEB problems in children, more intensive service delivery formats and service methods are needed; third, universities are good intervention settings for scientific research and professional specialties to implement and standardize the program more rigorously, thereby improving the effectiveness of interventions.

While Sanders et al. (2014) and Nowak and Heinrichs (2008) conducted comprehensive systematic reviews of Triple P literature, our research differs *via* the following aspects. First, the inclusion and exclusion criteria were different. By strictly adhering to the PICOS approach, our research had stricter requirements for the included studies, which were limited to RCTs and quasi-experimental studies with higher research quality, and, because of the focus of research design, we did not use *the study approach* as a moderating variable to analyze its impact on outcomes. Second, our research analyzed SEB problems in children separately, supported by the evidence that Triple P is effective in reducing emotional problems in children. Simultaneously, our research reported the different dimensions of parent outcomes separately to describe their specific connotations and manifestations more clearly. Third, in terms of the research purpose, our research was not limited to a comprehensive review of the effects of Triple P but also attempted to provide an analytical reference for more interventions of Triple P in other countries that have fewer studies available than Australia based on the results of the systematic review.

There are some limitations to our study. First, we should counteract the file drawer problem by simultaneously analyzing published and unpublished studies. However, we included only published studies in this research. Second, in terms of database retrieval, we only searched the journal databases and did not search for articles and dissertations related to Triple P in other ways. We will continue to expand the database in this area in future. Third, we only included new research but did not include past research, so we cannot give the full picture very well, particularly in terms of the moderator effects, with a low number of studies. We will conduct another meta-analysis including all previous research in the future. Last, there are still some potential risks of bias in-depth that need to be assessed and eliminated using Cochrane RoB 2.0 in the future, including the risk of bias within and across studies.

## Data Availability Statement

The raw data supporting the conclusions of this article will be made available by the authors, without undue reservation.

## Author Contributions

NL and JP conceived the study. NL, JP, and YL conducted the literature search, screened studies for eligibility, extracted and coded data from the initial studies, revised the manuscript, and approved the final version. JP wrote up the Introduction section. YL wrote up the Method section. NL performed the statistical analyses and wrote up the Results, Discussion, and Conclusion sections. All authors contributed to the article and approved the submitted version.

## Conflict of Interest

The authors declare that the research was conducted in the absence of any commercial or financial relationships that could be construed as a potential conflict of interest.

## Publisher's Note

All claims expressed in this article are solely those of the authors and do not necessarily represent those of their affiliated organizations, or those of the publisher, the editors and the reviewers. Any product that may be evaluated in this article, or claim that may be made by its manufacturer, is not guaranteed or endorsed by the publisher.
