# Supplementary material for: Effects and Moderators of Triple P on the Social, Emotional, and Behavioral Problems of Children: Systematic Review and Meta-Analysis
Source: Front Psychol. 2021 Aug 26;12:709851. doi: 10.3389/fpsyg.2021.709851 (PMC8427298; doi:10.3389/fpsyg.2021.709851)
Supplement: Supplementary file 1 [file Data_Sheet_1.PDF]

Appendix The specific measurement tools of outcomes

**Outcomes**	**Measurements**
Social Competence	① SDQ-prosocial behavior
Emotional Problems in Children	① SDQ-emotional symptoms<br>② CAPES-Emotional
Behavioral Problems in Children（total）	① ECBI：Eyberg Child Behaviour Inventory<br>② CBCL：Child Behavior Checklist<br>③ SDQ：Strengths and difficulties questionnaire: Parent Form<br>④ DBC-P：Developmental Behaviour Checklist–Primary Carer version<br>⑤ CAPES：Child Adjustment and Parent Efficacy Scale<br>⑥ APBQ：Academic Problem Behavior Questionnaire
Parenting Style	① PS-Parenting Scale<br>② PAFAS Parenting Scale：The Parenting and Family Adjustment Scales<br>③ APQ：Alabama parenting questionnaire<br>④ FAD-general functionality：Family Assessment Device<br>⑤ CRPR: The Child Rearing Practices Report
Conflict over Parenting	① PPC：Parenting Problem Checklist<br>② PARI：Parental Attitude Research Instrument<br>③ PAFAS：PAFAS-parental teamwork<br>④ AS：The Acrimony Scale
Parenting Confidence	① PTC：The Parenting Tasks Checklist<br>② PSOC：Parenting Sense of Competence Scale<br>③ CAPES-The Efficacy Scale<br>④ PSE：Parental Self-Efficacy
Parental Adjustment	① DASS：Depression Anxiety Stress Scale<br>② PSI：Parenting Stress Index<br>③ PAFAS-parental adjustment<br>④ SNQ stress：Service Needs Questionnaire<br>⑤ GHQ：The General Health Questionnaire
Relationship quality and Satisfaction of parents	RQI：The Relationship Quality Index
Parent-Child Relationship	① CBQ：Conflict Behavior Questionnaire<br>② PCQ：Parent Conflict Questionnaire<br>③ PAFAS Parenting Scale-Parent-Child Relationship
